# Functional Brain Connectivity in Mild Cognitive Impairment With Sleep Disorders: A Study Based on Resting-State Functional Magnetic Resonance Imaging

**DOI:** 10.3389/fnagi.2022.812664

**Published:** 2022-03-10

**Authors:** Yuxi Luo, Mengyuan Qiao, Yuqing Liang, Chongli Chen, Lichuan Zeng, Lin Wang, Wenbin Wu

**Affiliations:** ^1^Department of Geriatrics, Hospital of Chengdu University of Traditional Chinese Medicine, Chengdu, China; ^2^Department of Radiology, Hospital of Chengdu University of Traditional Chinese Medicine, Chengdu, China; ^3^Health Management Center, Sichuan Academy of Medical Sciences and Sichuan Provincial People’s Hospital, Chengdu, China

**Keywords:** default-mode network, precuneus, functional compensation, functional connectivity, mild cognitive impairment, sleep disorder

## Abstract

**Purpose:**

To investigate the effect of sleep disorder (SD) on the changes of brain network dysfunction in mild cognitive impairment (MCI), we compared network connectivity patterns among MCI, SD, and comorbid MCI and sleep disorders (MCI-SD) patients using resting state functional magnetic resonance imaging (RS-fMRI).

**Patients and Methods:**

A total of 60 participants were included in this study, 20 each with MCI, SD, or MCI-SD. And all participants underwent structural and functional MRI scanning. The default-mode network (DMN) was extracted by independent component analysis (ICA), and regional functional connectivity strengths were calculated and compared among groups.

**Results:**

Compared to MCI patients, The DMN of MCI-SD patients demonstrated weaker functional connectivity with left middle frontal gyrus, right superior marginal gyrus, but stronger connectivity with the left parahippocampus, left precuneus and left middle temporal gyrus. Compared to the SD group, MCI-SD patients demonstrated weaker functional connectivity with right transverse temporal gyrus (Heschl’s gyrus), right precentral gyrus, and left insula, but stronger connectivity with posterior cerebellum, right middle occipital gyrus, and left precuneus.

**Conclusion:**

Patients with MCI-SD show unique changes in brain network connectivity patterns compared to MCI or SD alone, likely reflecting a broader functional disconnection and the need to recruit more brain regions for functional compensation.

## Introduction

The incidence and prevalence of dementia are rising in many regions due to population aging. According to estimates by Alzheimer’s Disease International, more than 50 million people worldwide had dementia in 2019, and this number will increase to 152 million by 2050 (Lopez and Kuller, 2019). Dementia results in impairment of learning, memory, understanding, orientation, computation and other cognitive functions. With disease progression, the patient gradually loses the ability for self-care, placing a heavy burden on both the family and healthcare system. Alzheimer’s disease (AD) is the most common type of dementia, accounting for about 50–70% of all cases (Julie and Mary, 2014). While many AD risk genes have been identified and neurodegeneration strongly linked to the formation of extracellular plaques and intracellular neurofibrillary tangles (NFTs), there are still no broadly effective therapies. MCI is a borderline between normal aging and dementia and is widely considered a precursor to AD and other neurodegenerative disorders. Since MCI has the potential to remain stable and not deteriorate or even reverse into a normal cognitive state, it has become a goal to study the transition mechanism of MCI to AD and how to effectively prevent its further development.

SD almost always occur in people with cognitive impairment, and 25–40% of AD patients may have sleep disturbance in the early disease stages even before the appearance of cognitive dysfunction (Kaicheng et al., 2019). Patients with MCI are more than three times more likely to have overall poor sleep than cognitive healthy controls (Katie et al., 2018). A study of 431 patients with cognitive impairment found that patients with AD and MCI had almost the same frequency of any sleep disturbance (Guarnieri et al., 2012). SD associated with AD and MCI are manifested primarily as prolonged sleep onset latency/difficulty falling asleep, disturbances of the sleep-wake cycle and circadian rhythm, frequent nighttime waking (Guarnieri et al., 2012), and excessive daytime sleepiness (Congkang et al., 2013). Numerous studies have found that sleep disturbance and AD-associated cognitive impairments are mutually reinforcing, with sleep disturbance accelerating cognitive deterioration, and Aβ accumulation producing a direct negative effect on sleep function (Ju et al., 2014; Wang and Holtzman, 2020). Among the first neuropsychiatric deficits in AD is memory loss. Sleep helps to integrate and consolidate memories, so long-term SD can further impair memory (Hua and Yonghua, 2020). These strong reciprocal associations suggest shared pathomechanisms. Indeed, Osorio et al. (2014) found significant elevations of Aβ42, P-tau, and T-tau in cerebrospinal fluid (CSF) as well as reduced metabolic rate in the medial temporal lobe of sleep apnea patients. Others have reported that nocturnal awakenings were associated with Aβ burden in the precuneus (You et al., 2019). Moreover, nocturnal awakenings were found to be an intermediary between Aβ and cognitive impairment (You et al., 2019). A sustained decrease in sleep duration may also increase Aβ load in the angular gyrus, anterior and posterior cingulate, precuneus and frontal lobes (Sprecher et al., 2015). Qianqian et al. (2018) found that sleep deprivation exacerbated cognitive dysfunction, induced morphological changes to hippocampal neurons, and increased senile plaque formation in the hippocampus and temporal cortex of APP/PS1 double transgenic AD model mice. In addition, sleep loss also disrupts normal Aβ metabolism by affecting the lymphatic system, hypocretin system, and neuroinflammatory responses, ultimately leading to pathological damage (Krueger et al., 2016; Peng et al., 2016; Wu et al., 2019). Since sleep disorders can interact with each other and form a vicious cycle, the early intervention of sleep disorders can improve the cognitive status of patients, reduce the risk of AD and delay its progression.

RS-fMRI is a non-invasive neuroimaging method that can identify abnormalities in resting state brain networks associated with MCI and SD without specific task requirements. With the gradual maturity of the research techniques and methods of resting state, technical advances in RS-fMRI have demonstrated functional connectivity patterns associated with specific cognitive processes as well as abnormalities associated with neurological diseases. For instance, imaging of low-frequency blood oxygen signals from the whole brain combined with ICA has been used to distinguish among different resting state networks, such as the DMN, salience network (SN), and cognitive control network (CCN). The high synchronicity of activity among these brain regions suggests collective contributions to specific neurological functions (Damoiseaux et al., 2006). In the resting state, the brain is not processing external tasks, but a cluster of regions termed the “default mode network” remains active to support internal mental states and monitor the external environment to cope with unforeseen events (Greicius et al., 2004; Yu and Hua, 2014). A large number of previous RS-fMRI studies have found abnormal functional connectivity changes in the DMN of MCI and AD patients and strong associations between these changes and neuropsychiatric symptoms (Pan et al., 2017; McDonough et al., 2020).

Numerous studies have shown specific functional changes in the DMN among MCI and SD patients, but few have analyzed such network alteration in patients with comorbid MCI and sleep disorders. Therefore, this study compared DMN connectivity among MCI, SD, and MCI-SD patients to identify network alterations that may explain the strong association and interaction between these disorders. We hypothesized that MCI-SD patients would show disrupted connectivity among both memory- and sleep-related areas within resting state networks, and that these disruptions would be more severe than in MCI or SD alone. It is well-known that the hippocampus and its surrounding regions are important brain regions involved in the encoding of explicit memory. Previous studies have also shown that compared with healthy participants, the functional characteristics of the hippocampus and surrounding regions have significantly changed in MCI patients (Celone et al., 2006). In addition, as the main regions of the default network, precuneus, posterior cingulate cortex (PCC), and retrosplenial cingulate cortex (RSCC) are also closely associated with episodic memory or autobiographical memory (Fellgiebel et al., 2004; Cera et al., 2019). Moreover, evidence has supported characteristic changes of the precuneus, and cingulate cortex in functional patterns of MCI patient (Pihlajamäki et al., 2009; Cera et al., 2019). A meta-analysis suggested that the changes of brain functional connectivity in sleep disorders are correlated with the DMN, SN, CCN, and the affect network (AN) (Haixia et al., 2019). Their changes are not only within each network, but also interact with each other, showing dynamic characteristic functional connectivity changes with the development of sleep disorders. Within DMN network, it was mainly manifested in the medial prefrontal lobe, precuneus, posterior cingulate gyrus, and temporal lobe. Therefore, we hypothesized that significant changes in characteristic functions of the hippocampus and related peripheral regions, precuneus, PCC/ACC, temporal lobe, and prefrontal lobe would be mainly observed.

## Materials and Methods

### Participants

Participants were recruited from inpatients and outpatients of Sichuan Provincial People’s Hospital and Hospital of Chengdu University of Traditional Chinese Medicine. They were all right-handed and of Han nationality. All MCI patients met the following criteria: (1) Chief complaint of memory loss confirmed by family, or physician, (2) Objective impairment in memory or in one other area of cognitive function as evident by scores > 1.5 S.D. below the mean of the same age and education group, (3) Normal global cognitive function, and the cut-off points of Mini-Mental State Examination (MMSE) scores were 17 (illiterate), 20 (elementary school graduate), and 24 (junior high school graduate or above), (4) [Clinical Dementia Rating (CDR) = 0.5, 5] Normal or slightly impaired activities of daily living (ADL score ≤ 16/56 subscale), and (6) Absence of dementia.

All sleep disorder patients met the following criteria: (1) confirmed diagnosis according to Diagnostic and Statistical Manual of Mental Disorders (DSM-V), (2) Pittsburgh Sleep Quality Index (PSQI) score ≥ 8 (Xianchen et al., 1996), and (3) no obvious cognitive impairment or history of other mental and neurological diseases. Participants in the MCI-SD group met the inclusion criteria of both MCI and SD.

Exclusion criteria for all candidates were as follows: (1) other neurodegenerative diseases, brain trauma, or cerebrovascular diseases, (2) serious heart, liver, or renal insufficiency, (3) history of poorly controlled diabetes or other major metabolic diseases, (4) secondary insomnia caused by alcohol, drug dependence, physical disease, or schizophrenia, (5) HAMD score ≥ 7 (indicative of clinical anxiety), and (6) other sleep disorders such as sleep apnea syndrome and circadian rhythm sleep disorders. Finally, a total of 60 participants were included in this study, 20 each with MCI, SD, or 20 MCI-SD. All participants met the physical requirements of MRI examination. Each participant gave written informed consent to participate. Each of them was scheduled for imaging on the same day after completing the neuropsychological measurements. This study was approved by the Ethics Committee of Sichuan Provincial People’s Hospital and has been conducted in accord with the 1964 Helsinki declaration and its later amendments or comparable ethical standards.

### Data Acquisition

All images were acquired using a Siemens 3 T Magnetom Trio MRI. Participants were instructed to remain awake but with eyes closed and head still and try not to think of anything specifically. Structural images were obtained by T1 imaging in the sagittal plane using the following parameters: sagittal scanning, 176 slices, thickness/gap = 1.0/0 mm, field of view [FOV = 200 × 200 mm^2^, repetition time (TR) = 1,900 ms, echo time (TE) = 2.13 ms, and inversion time (TI) = 900 ms]. Resting state fMRI images were acquired by axial scanning using an echo-planar imaging (EPI) sequence as follows: slices = 33, TE = 30 ms, TR = 2,000 ms, flip angle = 90°, slice thickness = 3.5 mm, matrix = 64 × 64, and FOV = 200 × 200 mm^2^.

### Analysis of Resting State Functional Magnetic Resonance Imaging Data

#### Preprocessing

In this study, we focused on changes in the functional connectivity pattern of the DMN using RS-fMRI. Preprocessing of the RS-FMRI data was performed using SPM12 software^1^ running on the Matlab platform. Participants with head motion exceeding 3 mm in translation and 3° in rotation during the RS-fMRI scan have been eliminated. First, to eliminate signal instability at the beginning of the scan, all resting state DICOM image data were converted to *.hdr and format using the dcm2nii tool of MRIcron software.^2^ Then, the first 5 time points were eliminated. Standard preprocessing was performed on the remaining functional images, including head motion correction, slice timing correction, spatial normalization to the standard Montreal Neurological Institute (MNI) template with resampling to 3 × 3 × 3 mm^3^, and spatial smoothing using an 8 mm full-width at half-maximum isotropic Gaussian kernel. All smoothed images were then bandpass filtered (0.01–0.1 Hz) and detrended to reduce low-frequency drift and physiological high-frequency respiratory and cardiac noise. Finally, we selected Friston 24 to remove covariates, mainly the effects of head movement, whole brain signal, white matter signal and cerebrospinal fluid on low frequency synchronous oscillation signal.

#### Independent Component Analysis

After preprocessing, 25 independent components of the resting state data were analyzed using independent component analysis (ICA) software (GIFTversion4.0b).^3^ The main procedures consisted of (1) principal component analysis (PCI) of each subject dataset to reduce the number of dimensions, (2) ICA, and (3) data reconstruction. GIFT software was used to calculate and identify the components, and the number of components was 71. GIFT’s Display GUI module displayed all the components of all the participants, but no ideal brain network was extracted. According to prior knowledge and previous studies (Beckmann et al., 2005; Smith et al., 2009), we selected 25 components to extract the default mode network, and the appropriate brain functional network was obtained. Then, the same components from each subject were extracted to form sub-packets. Covariance analysis was used to compare differences in functional connectivity Z-scores among groups [*q* < 0.05, false discovery rate (FDR) corrected]. Functional networks were identified by spatially correlated program templates that matched standard templates, and visual checks were performed based on spatial topology, time series, and low-frequency content to exclude false networks arising from physiological and/or motor artifacts. The DMN was then selected for comparative analysis.

## Results

### Demographic Data and Pittsburgh Sleep Quality Index Score, Mini-Mental State Examination Score

There were no differences in mean age, sex ratio, and education level among MCI, SD, and MCI -SD groups (Table 1). As expected according to group stratification criteria, PSQI scores were markedly higher in the SD group (16.00 ± 1.17) and MCI-SD group (11.90 ± 2.80) compared to the MCI group (3.70 ± 1.03) (*P* < 0.01) (Table 2). The MMSE scores demonstrated significant differences among the three groups (*F*-test of equality of variances; *F* = 39.62, *p* < 0.001). Details are presented in Table 3.

**TABLE 1:** Demographics of Mild Cognitive Impairment (MCI), sleep disorder (SD), and comorbid MCI-SD groups.

	MCI	*SD*	MCI-SD	F/χ 2	*p*
Age (years)	66.35 (6.69)	66.50 (5.49)	66.85 (7.05)	0.03	0.97
Female sex (female: male)	35.00%	25.00%	35.00%	0.61	0.73
Educational level (years)	10.15 (3.23)	10.68 (1.82)	9.65 (2.47)	0.59	0.56

*Data are presented as mean ± standard deviation.*

**TABLE 2:** Sleep scores and subscores for each group.

	MCI	*SD*	MCI-SD	F	*p*
PSQI scores	3.70 (1.03)	16.00 (1.17)	11.9 (2.80)	228.14	< 0.01
Sleep quality score	0.85 (0.49)	2.45 (0.69)	1.90 (0.97)	25.71	< 0.01
Sleep latency score	0.40 (0.50)	2.85 (0.37)	1.90 (1.11)	55.84	< 0.01
Sleep time score	0.65 (0.67)	2.40 (0.60)	1.95 (0.51)	46.38	< 0.01
Sleep efficiency score	0.15 (0.37)	2.45 (0.83)	2.45 (0.83)	44.55	< 0.01
Sleep disturbance score	1.00 (0.46)	1.70 (0.47)	1.45 (0.51)	10.91	< 0.01
Used sleep medicine score	0.00 (0.00)	1.45 (1.27)	0.80 (1.23)	9.99	< 0.01
Daytime dysfunction score	0.65 (0.59)	2.70 (0.47)	1.80 (1.11)	35.45	< 0.01

*Data are presented as mean ± standard deviation. F, F-test of equality of variances.*

**TABLE 3:** MMSE scores for each group.

	MCI	*SD*	MCI-SD	*F*	*P*
MMSE	20.80 (2.74)	27.55 (1.54)	24.55 (2.72)	39.62	<0.001

*Data are presented as mean ± standard deviation. MMSE, Mini-Mental State Examination; F, F-test of equality of variances.*

### Intra-Group Connectivity

**FIGURE 1:**
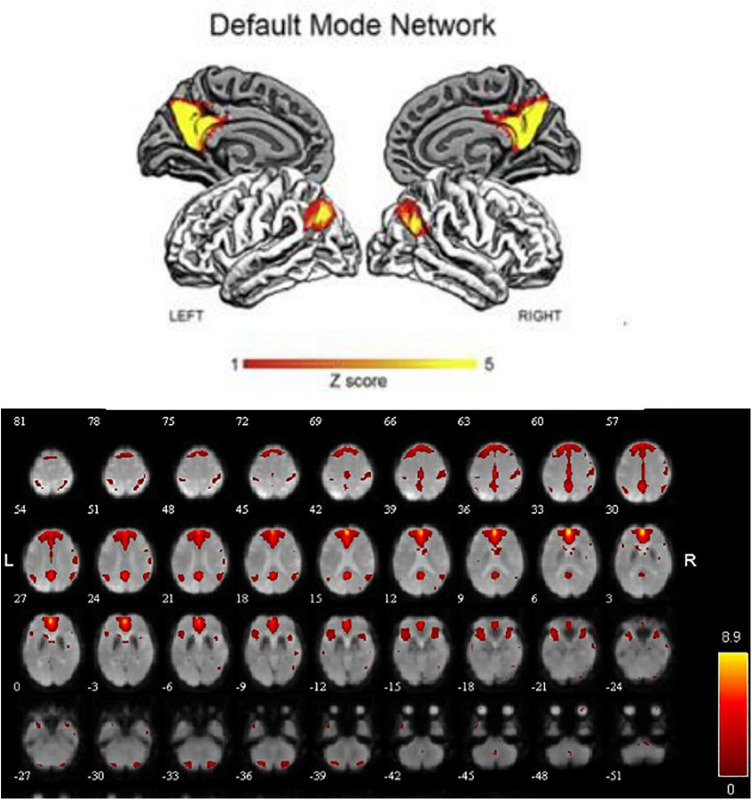
The default mode network distribution (red).

In order to explore the functional connectivity pattern of MCI-SD patients, we use ICA to select DMN, which mainly includes bilateral prefrontal lobes, bilateral temporal lobes, middle frontal gyrus, posterior cingulate gyrus, precuneus, and bilateral inferior parietal lobule (Figure 1).

**FIGURE 2:**
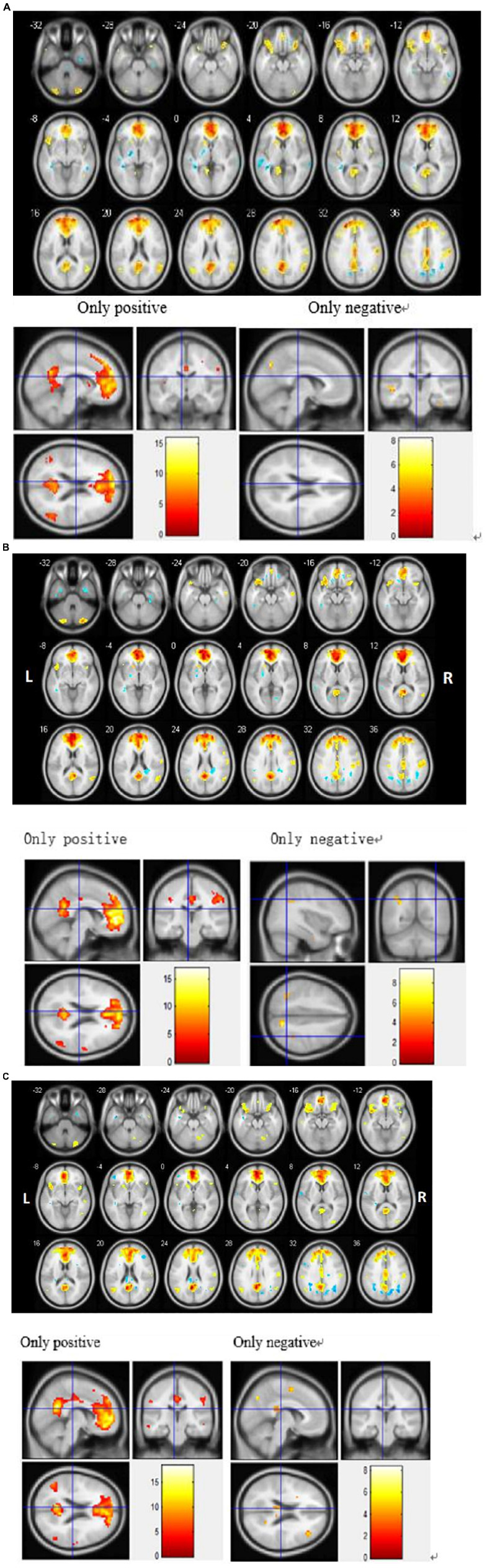
**(A)** Regions showing significant differences in default mode network (DMN) functional connectivity (FC) among Mild Cognitive Impairment (MCI) patients. Blue indicates negative FC and red indicates positive FC. The threshold was set at a corrected *p* < 0.001. The color bar indicates the *T*-value from within-group *t*-tests. **(B)** Regions showing significant differences in default mode network (DMN) functional connectivity (FC) among sleep disorder (SD) patients. Blue indicates reduced FC and red indicates increased FC. The threshold was set at a corrected *p* < 0.001. The color bar indicates the *T*-value from within-group *t*-tests. **(C)** Regions showing significant differences in default mode network (DMN) functional connectivity (FC) among patients with comorbid MCI and SD. Blue indicates reduced FC and red indicates increased FC. The threshold was set at a corrected *p* < 0.001. The color bar indicates the *T*-value from within-group *t*-tests.

To detect activation of the DMN at the cohort level (MCI, SD, and MCI-SD group), we performed a one-sample *T*-test on imaging data of the three groups. There were significant differences in functional connectivity values within all three groups [*P* < 0.001 by one-sample *t*-test, (FDR) corrected]. Group-level images of the DMN are presented in Figure 2. In the MCI group, the bilateral precuneus, bilateral anterior cingulate gyrus, bilateral internal frontal gyrus, left superior margin gyrus, right posterior central gyrus, and bilateral angular gyrus showed significantly positive connectivity, while the inferior parietal lobule, parahippocampus, and cerebellar tonsil exhibited significantly negative connectivity. In the SD group, bilateral precuneus, bilateral anterior cingulate gyrus, bilateral internal frontal gyrus, right superior temporal gyrus, right posterior central gyrus, and bilateral angular gyrus demonstrated significantly positive connectivity, while bilateral inferior parietal lobule and bilateral fusiform gyrus showed significantly negative connectivity. Finally, in the MCI-SD group, bilateral precuneus, bilateral anterior cingulate gyrus, bilateral inferior frontal gyrus, bilateral angular gyrus, and cerebellar tonsil showed positive connectivity, whereas left internal frontal gyrus, right middle temporal gyrus, and putamen demonstrated negative connectivity.

### Intergroup Comparison of Brain Functional Networks

**FIGURE 3:**
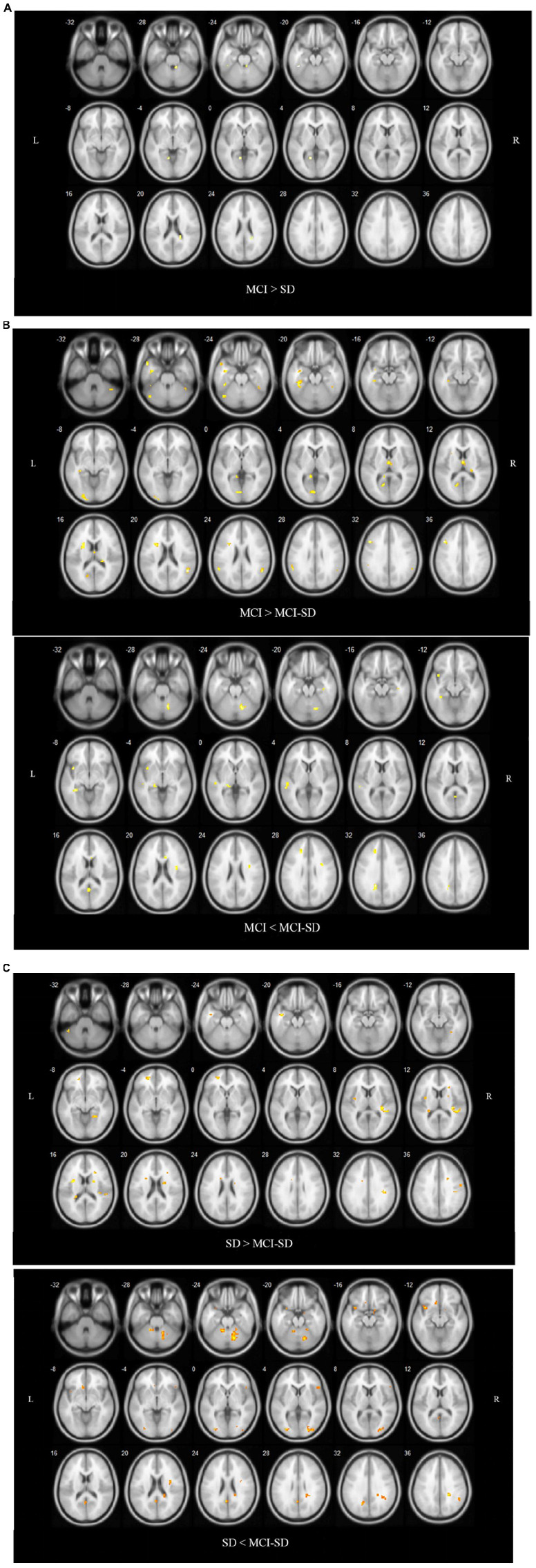
**(A)** Regions showing significant differences in DMN FC between the MCI group and SD group. Blue indicates reduced FC. The threshold was set at a corrected *p* < 0.001. **(B)** Regions showing significant differences in DMN FC between the MCI group and comorbid MCI-SD group. The top images show brain areas with stronger FC in the MCI group than the MCI-SD group (MCI > MCI-SD), while the lower images show brain areas with weaker FC in the MCI group (MCI < MCI-SD). The threshold was set at a corrected *p* < 0.001. FC, function connectivity. **(C)** Regions showing significant differences in DMN FC between the SD group and MCI-SD group. Images above show brain areas of the brain with stronger FC in the SD group (SD > MCI-SD), while lower images show areas with weaker FC in the SD group than the MCI-SD group (SD < MCI-SD). The threshold was set at a corrected *p* < 0.001. FC, function connectivity.

Covariance analysis was used to compare differences in brain functional networks between groups. Select one Way Anova in SPM, set F Contrast [1-1; 0 1-1] in contrast, and take age, gender, and educational level as covariates. These were also significant differences in functional connectivity among groups (*P* < 0.001 by Covariance analysis). Figure 3 and Table 4 show these paired comparison results. Compared to the SD group, the MCI group DMN exhibited stronger functional connectivity with the left inferior temporal gyrus and left lingual gyrus, while no DMN brain regions exhibited weaker connectivity. Compared to the MCI group, the MCI-SD group DMN exhibited weaker functional connectivity with the left middle frontal gyrus, right superior marginal gyrus, and stronger functional connectivity with the left parahippocampus, left precuneus, and left middle temporal gyrus. Finally, compared to the SD group, the MCI-SD group DMN demonstrated stronger functional connectivity with the posterior cerebellum, right middle occipital gyrus, and left precuneus, and weaker functional connectivity with the right transverse temporal gyrus (Heschl’s gyrus), right precentral gyrus, and left insula.

**TABLE 4:** Differences in functional connectivity among AD, SD, and AD-SD groups.

	MNI Coordinates			
	
Region	x	y	Z	*T*-value
MCI > SD				
Temporal_Inf_L	−45	−42	−21	4.68
Lingual_L	−12	−57	0	4.39
MCI > MCI-SD				
Temporal_Sup_R	51	−51	21	4.21
Frontal_Mid_L	−45	18	36	4.27
MCI < MCI-SD				
ParaHippocampal_L	−33	−39	−9	−3.32
Precuneus_L	0	−57	15	−3.17
Temporal_Mid_L	−57	−36	6	−4.01
SD > MCI-SD				
Heschl_R	36	−27	12	4.35
Insula_L	−33	0	15	3.78
Precentral_R	42	−18	48	5.16
SD < MCI-SD				
Occipital_Mid_R	27	−87	6	−4.24
Precuneus_L	−3	−54	24	−3.97
Cerebelum_6_R	15	66	−24	−4.31

*MNI, Montreal Neurological Institute. For each peak voxel x-, y-, and z-coordinates in the MNI-152 standard space image are given. L, left; R, right.*

## Discussion

In this study, we used ICA to extract the DMN, and the intra-group comparison was analyzed by one-sample *t*-test. The DMN of three groups all showed bilateral precuneus, bilateral anterior cingulate, and bilateral angular gyrus significantly positive activation. The MCI and SD groups additionally showed significant positive connectivity of bilateral medial frontal gyrus, right postcentral gyrus. These similarities in DMN alterations again suggest shared pathomechanisms between MCI and SD. We used covariance analysis to compare the differences of functional connectivity in DMN among the three groups. When compared with the MCI group, the MCI-SD group showed increased functional connectivity in the left precuneus, left parahippocampus, and left middle temporal gyrus, while when compared with the SD group, the functional connectivity of the left precuneus, right middle occipital gyrus, and posterior cerebellum were increased.

The precuneus, medial temporal lobe (including the hippocampus, parahippocampal gyrus, and the entorhinal cortex etc.), cingulate gyrus, and prefrontal lobe are currently considered to be important regions related to episodic memory (Yuan et al., 2020). The precuneus contributes to high-level cognitive functions, including episodic and working memory retrieval, attention, and conscious perception, through strong connectivity with subcortical structures of the limbic system, such as the hippocampus, amygdala, and thalamus (Lundstrom et al., 2005). Another part of the limbic system, the anterior cingulate gyrus, is involved in cognitive control, distribution of attention, emotion regulation, working memory, and reward response (Gröne et al., 2015). The angular gyrus is also involved in the processing of various cognitive information such as memory retrieval, attention, and theory of mind. In general, the left prefrontal lobe is mainly involved in encoding and storing memories, while the right prefrontal lobe is more involved in retrieving memories (Grossman et al., 2003). In elderly persons without cognitive impairment, the integrity and functional connectivity of the DMN decline with age (Dennis and Thompson, 2014). Changes to multiple DMN components may occur in the elderly brain over time and these changes may indicate a loss of functional regulation (Beason-Held et al., 2017). The functional changes in these brain regions may be reflected in the normal “aging” decline in cognitive functions such as memory and attention, but the specific pathological changes in MCI make this functional change pattern different. In the resting state, some DMN regions showed significant activation, such as the precuneus, anterior cingulate gyrus, parts of the prefrontal cortex. It may reflect impairments in memory, cognition, and other functions in the three groups, which were compensated by compensatory mechanisms in the brain. Compensatory neuroplasticity allows one region to assume the functions of another following damage, and this process may require alterations in regional functional connectivity (Daniela et al., 2018). The degree of cognitive disability from neurodegenerative diseases depends in part on the amount of cognitive reserve accrued through education, ongoing intellectual activities, and social activities (Stern and Barulli, 2019). The precuneus, anterior cingulate, and middle frontal gyrus are thought to be associated with cognitive reserve (Stefano et al., 2016). Education, intellectual activities, and social activities are negatively correlated with resting state metabolic activities and/or cerebral blood flow in different cortical and subcortical regions. Stefano et al. (2016) found that elderly AD patients showed greater activation in brain regions related to cognitive reserve compared to healthy elderly people, suggesting compensation by other memory-related brain regions. A study by Yuan et al. (2020) found that with the development of the disease and the damage to the frontal lobes, the brain may exhibit compensatory redistribution of remaining lobar function. They observed that nodular centrality in the left middle frontal gyrus and left superior medial frontal gyrus was significantly higher in the AD group than in the MCI or HC groups. The centrality of left middle frontal gyrus nodules in the mild cognitive impairment (MCI) group was significantly higher than that in the severe cognitive impairment group. Decreased functional connectivity in the posterior default mode network and increased anterior and abdominal default mode network were observed in early AD, and connectivity in all regions decreased significantly as the disease progressed to the middle and late stages of AD, which supports the theory that the early compensatory effect occurred within the DMN (Damoiseaux et al., 2012). Compensatory effects also occur in MCI, and many studies have found a general decrease in DMN functional connectivity in MCI patients, or decreased activation at resting state, such as in the hippocampus and lateral parietal cortex (Farràs-Permanyer et al., 2015), and the cuneus/precuneus (Pan et al., 2017), but increased resting state activation in these and other regions has also been observed (Farràs-Permanyer et al., 2015). Reduced functional connectivity is thought to be associated with memory decline, while increased activity could be a compensation for damage by recruitment of other regions (Li et al., 2018). Some studies have suggested that compensatory effects may also occur in the elderly without neurodegenerative diseases, showing increased activation in the anterior brain areas and decreased activation in the posterior DMN areas, and proposed a “Posterior Anterior Shift in Aging (PASA)” model. However, some scholars put forward the model is not suitable for explaining all findings, because some older subjects failed in generating anterior DMN areas compensatory (Katell et al., 2011).

In addition to the common characteristics mentioned above, the following diversities are also worth noting. First, the parahippocampus, cerebellar tonsil, and inferior parietal lobule in the MCI group showed lower activation than other brain regions in the DMN network. However, no significant differences in the relevant regions were observed between the SD group and the MCI-SD group, and when compared with MCI, the MCI-SD group showed stronger functional connectivity in the left parahippocampus and the left precuneus. According to previous studies (Kaboodvand et al., 2018), the precuneus is considered to be an important gateway between the medial temporal lobe and other parts of the cerebral cortex involved in episodic memory. MMSE scores showed less cognitive impairment in the MCI-SD group than in the MCI group. Therefore, the enhanced functional connectivity between the left parhippocampal and left precuneus may contribute to the preserved cognitive function in the MCI-SD group. Since this study did not measure the correlation between MMSE and FC and the left region, the speculation needs careful consideration. Because DMN remains active in the resting state to consolidate the contents of memory (Buckner and DiNicola, 2019), functional connectivity may increase compensatively to make up for the corresponding loss of memory function in the case of impaired white matter (Acosta et al., 2019). It is also worth noting that when making comparation within MCI-SD group, we observed asynchronous activation of bilateral precuneus and right middle temporal gyrus, implying a separation of functional connectivity between them. The inferior temporal cortex (including the middle temporal gyrus and the inferior temporal gyrus) was inhibited as a repository of long-term visual memory in MCI patients, whereas healthy controls tended to show stronger interaction between the right inferior temporal cortex and other regions of the DMN (Zhong et al., 2014). We noticed the same thing in the MCI-SD group. However, when compared with the MCI group, the MCI-SD group demonstrated enhanced functional connectivity between the left middle temporal gyrus, the precuneus and the parhippocampal gyrus. In combination with the preceding analysis, we hypothesized that the enhanced functional connectivity was also intended to compensate for the functional loss of the right inferior temporal cortex. We can’t really tell if this compensation pattern exists in MCI patients because we don’t have a healthy control group. However, it is possible that the MCI-SD group needs to recruit more regions for functional compensation.

A study by Kaicheng et al. (2019) on the interaction of SD and AD *in vivo* proposed a “bipolar model” of the Alzheimer’s disease spectrum. The model suggests that with the progression of cognitive impairment, brain functional connectivity appears as compensatory effects of MCI and decompensated effects of AD (Kaicheng et al., 2019). The brain has certain neuroplasticity and functional compensation mechanism. When the damage is mild, the brain or DMN may guarantee the realization of daily functions through functional reorganization. With the further aggravation of cognitive damage, the compensation mechanism is gradually unable to be sustained, and eventually progress to the “decompensated effect” of AD patients (Gabriel et al., 2021). We speculate that sleep disorders may accelerate this decompensation process, that is, MCI patients with sleep disorders may show more decreased activation of DMN brain regions or a more extensive compensatory effect than MCI patients. As observed in this study, the MCI-SD group showed more extensive disruption of internal functional connectivity in the DMN and more compensatory effects than that in the MCI group when the cognitive function was more preserved. This may be explained by the fact that MCI patients with sleep disorders “overdraw” the compensatory capacity of these brain regions earlier, and therefore, may fall into the abyss of “decompensation” faster.

Second, when compared with the MCI or SD groups, DMN in the MCI-SD group showed positive activation in the bilateral inferior frontal gyrus and negative connectivity in the left internal frontal gyrus, indicating greater separation of functional synchronization within the prefrontal lobe. In the meantime, the MCI-SD group seemed to have reduced functional connectivity between the left middle frontal gyrus, right superior marginal gyrus, and left calcarine sulcus when compared to the MCI group. Previous studies have suggested that posterior DMN (including precuneus, posterior cingulate cortex, and lateral parietal cortex) shows decreased connectivity in the early stages of AD, while anterior DMN (concentrated in the medial prefrontal cortex) shows increased connectivity and decreases with disease progression (Jiayue et al., 2019). Under such a premise, the reduced functional connectivity in the MCI-SD group between prefrontal cortex and the post-DMN region may be interpreted as SDs accelerated the disconnectivity progress in MCI patients. This conjecture needs to be further proved in a larger sample.

Thirdly, there were significant differences between the MCI-SD group and the MCI group or the SD group. In brief, the regions with significant differences in functional connectivity were concentrated within the DMN in the MCI-SD group compared with those of the MCI group; while compared with the SD group, the functional connectivity between DMN and external brain regions showed significant differences (In the analysis, we mainly focused on DMN, but when using ICA to extract DMN, the extracted brain network covered a small part of the regions outside DMN, so we observed such a result). The functional connectivity between the precuneus and the right middle occipital gyrus was increased. The occipital lobe mainly processes visual information, and its damage to the occipital lobe will lead to the decline of visual-spatial and executive abilities (Bilo et al., 2013). An fMRI-based study of patients with primary insomnia found reduced functional connectivity between the middle occipital gyrus and the posterior cingulate gyrus, which are considered related to reduced daytime executive function in these patients (Yongli et al., 2014). This result seems to be contrary to that of the present study. However, due to the different reference participants and the small sample size of this study, it is unclear whether these functional connectivity differences truly reflected the specific brain functional patterns in MCI-SD patients, which still needs further studies. In addition, functional connectivity between the precuneus and posterior cerebellum was enhanced in the MCI-SD group. Another study found that SD also disrupt functional connectivity within the cerebellum and between cerebellum and its functional connectivity to DMN (Yanrui et al., 2019). Anatomy and imaging have found that the majority of the cerebellum link to cerebral association networks important to cognition. Some studies have also observed that the cerebellum is related to cognitive, emotional, and behavioral control, although the specific mechanism of how the cerebellum participates in it needs further research (Rapoport et al., 2000; Buckner, 2013). Therefore, we assumed that the enhanced functional connectivity between precuneus and posterior cerebellar lobes in MCI-SD was attributed to the compensatory mechanism of the cognitive impairment, and explained the sleep-related impairment in the MCI-SD group to some extent. However, there were significant differences in other sleep-related brain regions of the MCI-SD group. A decreased functional connectivity between the left insula and the right postcentral gyrus was examined in the MCI-SD group with that of the SD group. The insula is a complex region that involved in cognitive control processes, sensorimotor, and affective processes by making extensive connections to other brain functional regions (Uddin et al., 2017). The insula has been proposed to integrate external sensory information with internal signals of emotional and physical states, and plays a crucial role in emotional experience and subjective feelings, all of which have been proposed to play a role in insomnia (Chen et al., 2014; Uddin et al., 2017). The postcentral gyrus is the core region of the CCN, that is responsible for senior cognitive tasks like working memory and selective attention (Niendam et al., 2012). Patients with long-term insomnia also presented a reduced functional activity in this brain region (Dai et al., 2014). Taken together, the abnormal functional connectivity between the insula and the cerebellum in the MCI-SD group were not only affected by sleep disorders, but also the result of MCI-related cognitive impairment.

## Conclusion

Patients with comorbid MCI and SD exhibited more extensive changes in functional connectivity among DMN regions than patients with SD or MCI alone, possibly reflecting greater disruption of normal pathways and compensatory remodeling of network organization. Moreover, patients with MCI-SD may tend to recruit more compensatory function at the stage of relative retention of cognitive function. These changes were mainly manifested in enhanced compensatory functional connectivity in brain regions associated with cognitive impairment such as precuneus, anterior cingulate gyrus, angular gyrus, and parahippocampus, as well as impaired functional connectivity in sleep-related regions such as anterior central gyrus, insula, and cerebellum. In addition, MCI-SD patients also showed extensive separation of functional connectivity pathways among the internal frontal lobe, internal occipital lobe, temporal lobe, and cerebellum compared to patients with MCI alone.

This study still has many limitations, including the following aspects. First, our sample size is relatively small, which may produce a large sample bias. Besides, our results must be interpreted with some caution. Although both MCI and SD groups were taken as controls in the present study, a healthy control group would make our analysis of altered brain connectivity patterns in MCI-SD patients with sleep disorder more convincing. Subsequent study designs can be improved on this basis, and further statistical analysis of the connectivity between cognitive function score and brain function can be conducted. Thirdly, since MCI can be transformed not only into AD, but also into other types of dementia, we selected patients with MCI in general and did not explore them in depth, such as amnestic MCI and non-amnestic MCI. A longitudinal study found that MCI patients who progressed to AD 2–3 years later had a more severe decline in functional connectivity than MCI patients whose disease remained stable (Petrella et al., 2011). Predominantly memory decline aMCI was also different from non-amnestic mild cognitive impairment (MCI-SD) in terms of functional connectivity (Hafkemeijer et al., 2012). Lastly, different sleep disorders can be evaluated more objectively. Sleep disorder is a broad definition, including seven major categories that include insomnia disorders, sleep-related breathing disorders, central disorders of hypersomnolence, circadian rhythm sleep-wake disorders, sleep-related movement disorders, parasomnias, and other sleep disorder (Sateia, 2014). In this study, only the PSQI was used to assess the sleep quality of the three groups of participants. Follow-up studies could analyze the effects of different sleep disorders on the changes in brain functional connectivity in Alzheimer’s patients.

## Data Availability Statement

The original contributions presented in the study are included in the article/supplementary material, further inquiries can be directed to the corresponding author/s.

## Ethics Statement

The studies involving human participants were reviewed and approved by the Ethics Committee of Sichuan Provincial People’s Hospital. The patients/participants provided their written informed consent to participate in this study.

## Author Contributions

CLC contributed to the conception of the study. LCZ performed the experiment. LW performed the data analyses. YQL helped perform the analysis with constructive discussions. YXL and MYQ contributed significantly to analysis and manuscript preparation. WBW contributed to the final approval of the version and accountable for all aspects of the work. All authors contributed to the article and approved the submitted version.

## Conflict of Interest

The authors declare that the research was conducted in the absence of any commercial or financial relationships that could be construed as a potential conflict of interest.

## Publisher’s Note

All claims expressed in this article are solely those of the authors and do not necessarily represent those of their affiliated organizations, or those of the publisher, the editors and the reviewers. Any product that may be evaluated in this article, or claim that may be made by its manufacturer, is not guaranteed or endorsed by the publisher.

## Footnotes

1
http://www.fil.ion.ucl.ac.uk/spm/

2
http://www.nitrc.org/projects/mricron/

3
http://mialab.mrn.org/software/gift/
